# First Report and Phylogenetic Analysis of Mitochondrial Genomes of *Chrysomya villeneuvi* and *Sarcophaga genuforceps*

**DOI:** 10.3390/insects16010026

**Published:** 2024-12-29

**Authors:** Fengqin Yang, Jiao Xiao, Xiangyan Zhang, Yanjie Shang, Yadong Guo

**Affiliations:** Department of Forensic Science, School of Basic Medical Sciences, Central South University, Changsha 410013, China; may8515@163.com (F.Y.); 216506003@csu.cn (J.X.);

**Keywords:** mitochondrial genomics, *Chrysomya villeneuvi*, *Sarcophaga genuforceps*, phylogenetic analysis

## Abstract

The mitochondrial genome, highly conserved across species, is crucial for species identification, phylogenetic analysis, and evolutionary research. *Chrysomya villeneuvi* and *Sarcophaga genuforceps*, two species with significant forensic value, have been understudied in terms of genetic data. In this study, the complete mitochondrial genomes of *C. villeneuvi* (15,623 bp) and *S. genuforceps* (15,729 bp) were sequenced and analyzed. All thirteen protein-coding genes (PCGs) exhibited Ka/Ks ratios below one, indicating purifying selection and supporting their utility as barcoding markers. Phylogenetic analysis and genetic distance calculations based on PCGs showed that *C. villeneuvi* is closely related to *Chrysomya rufifacies* and *Chrysomya albiceps*, and *S. genuforceps* aligns more closely with *Sarcophaga kentejana* and *Sarcophaga schuetzei*. This research is the first to provide mitochondrial genome data for *C. villeneuvi* and *S. genuforceps*, expanding the genetic resources available for Calliphoridae and Sarcophagidae and offering a foundation for further forensic and evolutionary studies.

## Simple Summary

*Chrysomya villeneuvi* and *Sarcophaga genuforceps* hold significant forensic value, yet their mitochondrial genome structures remain unexplored and phylogenetic insights are lacking. This study sequenced the complete mitochondrial genomes of both species, providing detailed analyses of their genome composition, codon usage patterns, and evolutionary rates. Additionally, phylogenetic analysis was conducted to elucidate their genetic relationships, offering valuable insights into their evolutionary context.

## 1. Introduction

The mitochondrial genome of insects is a circular double-stranded DNA molecule, typically 14,000–20,000 bp in length. It generally comprises 13 protein-coding genes (PCGs), 24 RNA-coding genes (22 tRNA genes and two rRNA genes), and a non-coding control region [1]. Advances in high-throughput sequencing technology have significantly accelerated mitochondrial genome research, leading to an increasing number of published mitochondrial genomes. As a molecular marker, the mitochondrial genome is widely utilized in species identification, phylogenetic analysis, and population genetics due to its conserved organization and rapid evolutionary rate. For instance, cytochrome oxidase c subunit I (*cox1*) and cytochrome b (*cytb*) genes are used as barcoding markers for insect species identification [2], effectively addressing challenges in morphological-based identification. Additionally, mitochondrial DNA (mt DNA) serves as a powerful tool in phylogenetic studies, helping researchers understand genetic relationships among organisms [3] and enabling accurate assessments of taxonomic placement and evolutionary history across diverse species.

Blow flies and flesh flies are critical components of carrion insect communities and frequently appear at death scenes, where their life cycles can be used to estimate the postmortem interval (PMI) [4,5]. Within these groups, the families Calliphoridae (blowflies) and Sarcophagidae (fleshflies) are particularly noteworthy, with significant forensic value. *Chrysomya villeneuvi* Patton (Calliphoridae) has been primarily studied in Thailand and Malaysia, focusing on species identification, morphological characterization, and basic developmental data collection [6,7,8]. *Sarcophaga genuforceps* Thomas (Sarcophagidae) is widely distributed in southern China [9,10] but has received little attention in the literature. Despite their forensic significance, no complete mitochondrial genome sequences or phylogenetic analyses have been reported for *C. villeneuvi* and *S. genuforceps*. This lack of genetic and evolutionary data hinders a comprehensive understanding of their evolutionary relationships within Calliphoridae and Sarcophagidae. Furthermore, it limits the potential application of mitochondrial genes for accurate species identification in forensic research. Expanding genetic data resources for these species is essential to address these gaps and enhance their utility in forensic and evolutionary studies.

This study provides the first complete mitochondrial genome sequences and annotations for *C. villeneuvi* and *S. genuforceps*, along with a detailed analysis of their mitochondrial genome structures. As of now, the GenBank database includes mitochondrial genome data for 11 species of the genus *Chrysomya* and 52 species of the genus *Sarcophaga*, including the species newly reported in this study. For our analysis, we utilized the complete mitochondrial genome data available for all *Chrysomya* species and selected an equal number of *Sarcophaga* mitochondrial genomes. Phylogenetic trees were constructed to elucidate the evolutionary relationships among these species and their related taxa. This work aims to offer a foundational resource for future research employing mitochondrial genome data.

## 2. Materials and Methods

### 2.1. Sample Collection and DNA Extraction

The adult specimens of *C. villeneuvi* and *S. genuforceps* were captured on August 2020 in GaoPo Township, Huaxi District (26°43′ N, 106°68′ E), and Guiyang Forest Park (26°33′ N, 106°45′ E), which both are located in Guizhou Province, China. Samples were stored at −80 °C in Guo’s lab (Changsha, China). Total DNA from the thorax region of the flies was extracted using the QIANamp Micro DNA Kit (Tiangen Biotech Co., Ltd., Beijing, China) according to the manufacturer’s instructions.

### 2.2. Sequencing, Assembly, and Annotation of mt DNA

Sequencing was performed by OE Biotech. Co., Ltd. (Shanghai, China). A 250 bp DNA library was constructed according to the manufacturer’s protocol for Illumina^®^TruSeq^®^DNA samples and sequenced using an Illumina Hiseq 2500 platform (Illumina, Inc., San Diego, CA, USA) with a read length of 150 bp. Mitochondrial genome assembly was performed using MITObim v1.9 (https://github.com/chrishah/MITObim, accessed on 28 December 2024) [11]. The mitochondrial genomes were annotated using MITOS Web Server (http://mitos2.bioinf.uni-leipzig.de/index.py, accessed on 28 December 2024) [12], and the specific parameter was set to based code: 5—inverterbrate; reference: 89 RefSeq Metazoa. The secondary structure of tRNA genes was identified using tRNAscan-SE (https://lowelab.ucsc.edu/tRNAscan-SE/, accessed on 28 December 2024) [13]. The gene boundaries of 13 PCGs, 2 rRNAs, and 22 tRNAs were manually corrected though sequence alignment with other released reference mitogenomes of flies in GenBank. The annotated mitochondrial genome sequences were uploaded to NCBI and the obtained GenBank accession numbers are MW592365.1 (*C. villeneuvi*) and MW592364.1 (*S. genuforceps*).

### 2.3. Analysis of mt DNA

The graphical mitogenome circular maps of *C. villeneuvi* and *S. genuforceps* were drawn using Proksee (https://proksee.ca/projects/new, accessed on 28 December 2024) [14]. The invertebrate mitochondrial codon table was applied. Nucleotide composition and skewness levels, relative synonymous codon usage (RSCU), and amino acid frequencies of their PCGs were calculated in MEGA 11 [15]. AT-skew and GC-skew were calculated using the following formulas: AT-skew = [A − T]/[A + T] and GC-skew = [G − C]/[G + C]. The non-synonymous (Ka) and synonymous (Ks) substitutions of 13 PCGs within 11 Calliphoridae mitogenomes and 11 Sarcophagidae mitogenomes were calculated using DnaSP v6.0 [16]. The visualization was performed using GraphPad Prism v9.5.1 for Mac (GraphPad Software, San Diego, CA, USA, www.graphpad.com, accessed on 28 December 2024). The tRNA secondary structure visualization was performed in ViennaRNA Web Services/forna (http://rna.tbi.univie.ac.at/forna/, accessed on 28 December 2024) [17].

### 2.4. Phylogenetic Analyses

A total of 13 PCGs of 26 species (Table 1) were involved in phylogenetic analyses, including 11 *Chrysomya* mitogenomes, 11 *Sarcophaga* mitogenomes, 3 *Calliphora* mitogenomes, and 1 *Musca* mitogenome. The mitogenomes of *Calliphora nigribarbis*, *Calliphora chinghaiensis*, *Triceratopyga calliphoroides*, and *Musca domestica* were determined as the outgroup taxon. All mitogenome sequences used for phylogenetic analyses were aligned using MAFFT v7.490 [18]. The maximum likelihood (ML) phylogenetic tree was constructed through IQ-TREE 1.6.12 under the best-fit model GTR+F+R3 with the parameter (-bb 1000) [19]. The Bayesian inference (BI) phylogenetic tree was constructed using MrBayes 3.2.7a [20], employing the following command: mcmcp append = no ngen = 1,000,000 printfreq = 1000 samplefreq = 1000 nchains = 10 nruns = 4 savebrlens = yes checkpoint = yes checkfreq = 5000. BI results were considered reliable when the average standard deviation of split frequencies (ASDSF) was below 0.01, the potential scale reduction factor (PSRF) was close to 1, and the estimated sample size (ESS) exceeded 200. Once these requirements were met, the results were output for further analysis. Bootstrap values (BSs) and posterior probabilities (PPs) were calculated in ML and BI analysis, respectively. The tree visualization, modification, and annotation were performed using tvBOT v2.6 (https://www.chiplot.online/tvbot.html, accessed on 28 December 2024) [21].

In addition, evolutionary analyses were conducted in MEGA 11 [15]. This analysis was conducted using the Kimura 2-parameter model [22], and all ambiguous positions were removed for each sequence pair (pairwise deletion option). The visualization of the genetic distance was performed using GraphPad Prism v9.5.1 for Mac (GraphPad Software, San Diego, CA, USA, www.graphpad.com, accessed on 28 December 2024).

## 3. Results

### 3.1. Mitogenome Organization and Base Composition

**Figure 1:**
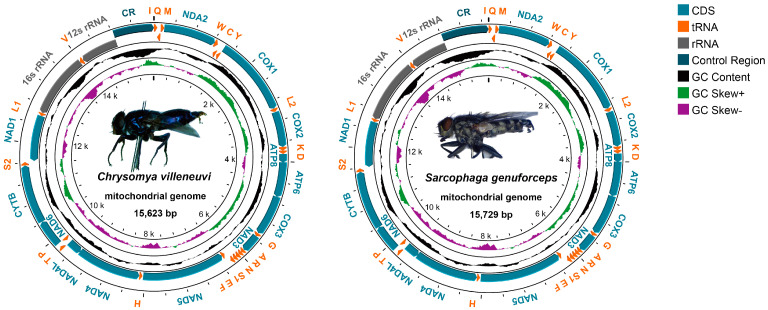
Circular mitogenome maps of *Chrysomya villeneuvi* and *Sarcophaga genuforceps*. The internal values represent the size of the mitogenome. The inner and outer loops where CDS, tRNA, rRNA, and the control region are located represent the L strand (−) and H strand (+), respectively.

The whole mitogenome sequences of *C. villeneuvi* and *S. genuforceps* are 15,623 bp and 15,729 bp in length, respectively. As shown in Figure 1 and Table 2, both species possess 37 genes, comprising 13 protein-coding genes (PCGs), 22 transfer RNAs (tRNAs), two ribosomal RNAs (rRNAs), and one control region. The non-coding control region, located between 12S rRNA and *trnI*, measures 426 bp in *C. villeneuvi* and 915 bp in *S. genuforceps*. Nine PCGs and 14 tRNAs are located on the heavy strand (+), while the remaining genes are situated on the light strand (−). Analysis of the mitochondrial genome structure revealed 12 overlapping genes in *C. villeneuvi* and 14 overlapping genes in *S. genuforceps*, with overlap lengths ranging from 1 to 9 bp. The longest gene overlap in both species occurs between the *trnW* and *trnC* genes, spanning 9 bp. Additionally, *C. villeneuvi* exhibits an overlap between *trnC* and *trnY* genes of 20 bp, and *S. genuforceps* shows an overlap between *trnE* and *trnF* genes of 20 bp.

The nucleotide composition analysis results (Table 3) showed that the mitochondrial base composition of *C. villeneuvi* and *S. genuforceps* was biased toward A and T. The AT-skew values were 0.03 and 0.04, and the GC-skew values were −0.18 and −0.19, respectively.

### 3.2. PCGs and RSCU Analysis

**Figure 2:**
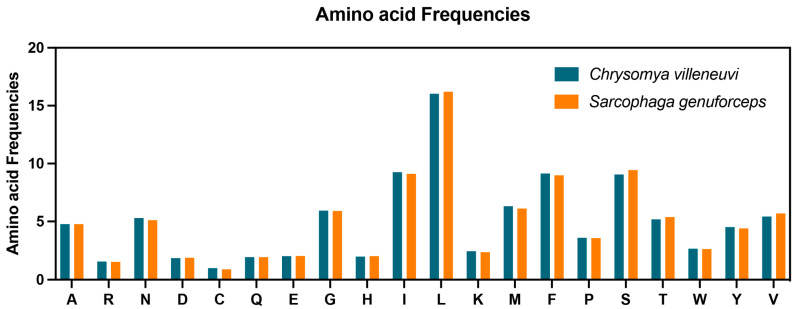
Amino acid frequencies of the mitochondrial PCGs of *Chrysomya villeneuvi* and *Sarcophaga genuforceps*.

**Figure 3:**
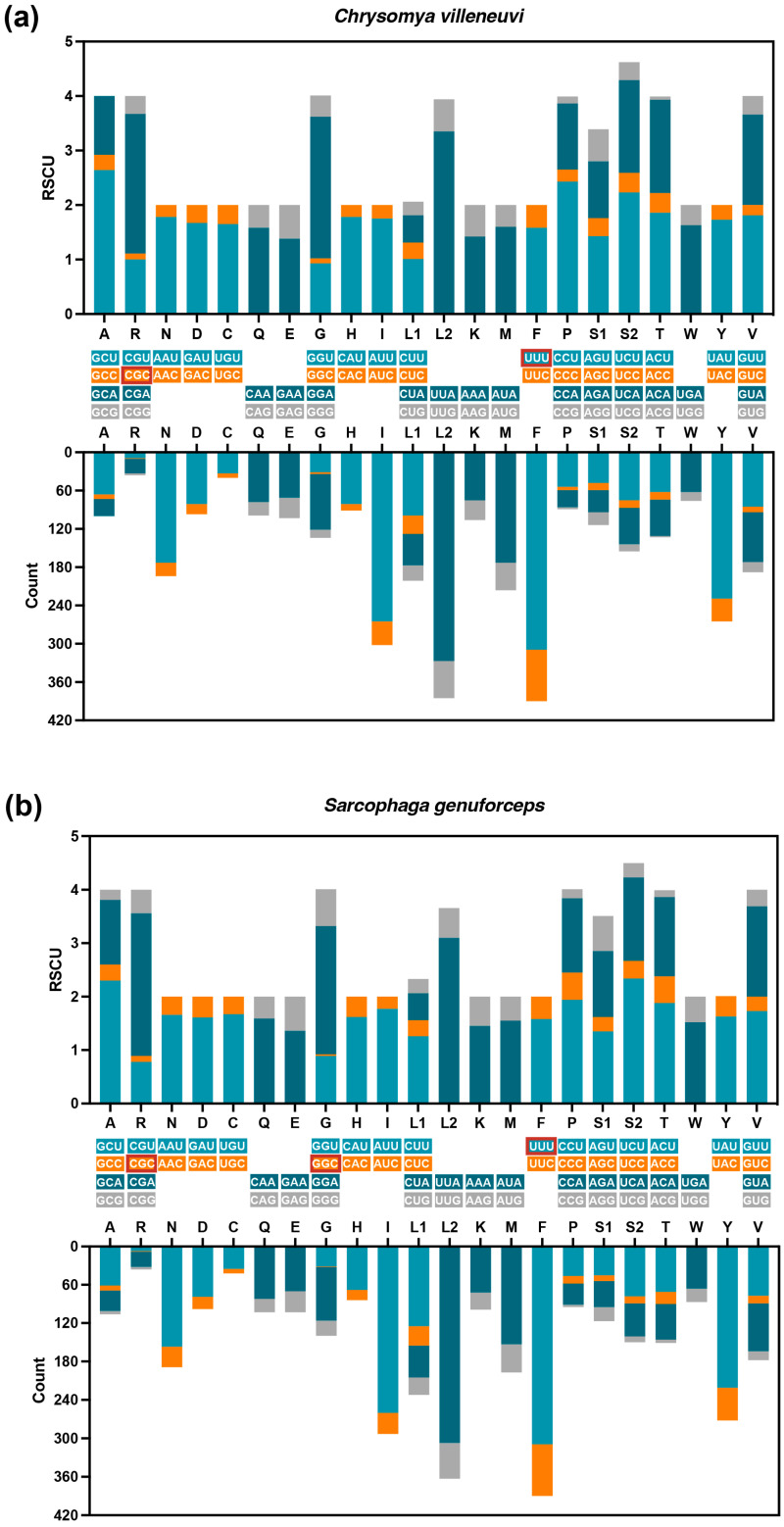
Relative synonymous codon usage (RSCU) and codon usage of *Chrysomya villeneuvi* (**a**) and *Sarcophaga genuforceps* (**b**). The red frame highlights the most and least frequently used codons in both species.

As shown in Table 2, the 13 PCGs of *C. villeneuvi* and *S. genuforceps* generally use ATT, ATG, and ATC as the start codon, except for *cox1* and *nad1*, which use TCG and TTG, respectively. The *atp8* start codon in *C. villeneuvi* is ATT, while in *S. genuforceps*, it is ATC. Stop codons are consistent across most PCGs, except for *cob*, which ends in TAA in *C. villeneuvi* and TAG in *S. genuforceps*. In genes with the same stop codon, an incomplete T serves as the stop codon for *cox1*, *cox2*, *nad4*, and *nad5*, while TAA is used for the remaining PCGs. The amino acid frequencies of PCGs (Figure 2) show that leucine (Leu, L) is the most common, accounting for 16.02% in *C. villeneuvi* and 16.21% in *S. genuforceps*, whereas cysteine (Cys, C) is the least common, at 0.99% and 0.89%, respectively. According to the RSCU analysis (Figure 3), UUU is the most frequently used codon in both species, occurring 309 times. The least frequently used codons are CGC in *C. villeneuvi* and CGC and GGC in *S. genuforceps*, each occurring once.

**Figure 4:**
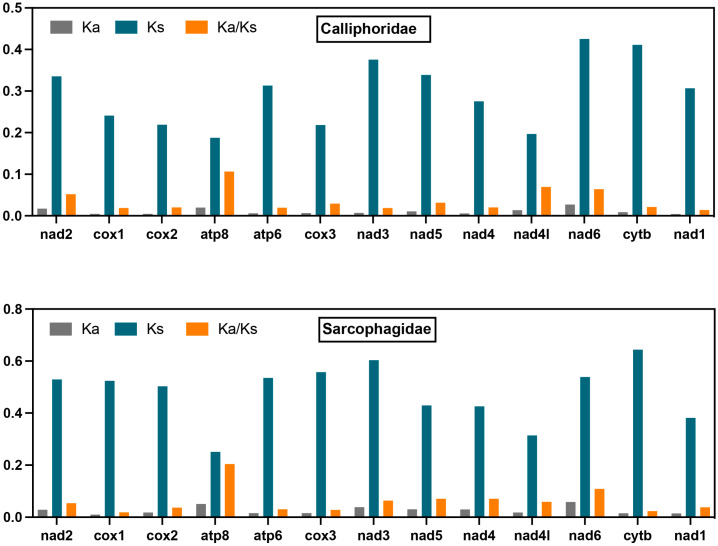
The evolutionary rate analyses of 13 protein-coding genes (PCGs) of the mitochondrial genome of 11 species in the Calliphoridae family and 11 in the Sarcophagidae family.

The Ka, Ks, and Ka/Ks values (Figure 4) of the 13 PCGs across 11 species in the Calliphoridae family and 11 in the Sarcophagidae family were calculated to investigate the relative evolutionary rates. The Ka/Ks ratios for all PCGs were below one, suggesting that these genes are under strong purifying selection, making them suitable as genetic markers. Among the PCGs, *atp8* showed the highest Ka/Ks ratios (0.11 in Calliphoridae and 0.20 in Sarcophagidae), indicating relatively higher evolutionary rates. The *nad1* (0.01), *nad3* (0.02), and *cox1* (0.02) in Calliphoridae and *cox1* (0.02), *cytb* (0.02), and *cox3* (0.03) in Sarcophagidae exhibited the lower Ka/Ks ratios.

### 3.3. tRNA and rRNA Genes

The total lengths of the tRNA and rRNA regions in the mitochondrial genomes of *C. villeneuvi* and *S. genuforceps* are 1469 bp and 2112 bp, respectively (Table 3).

**Figure 5:**
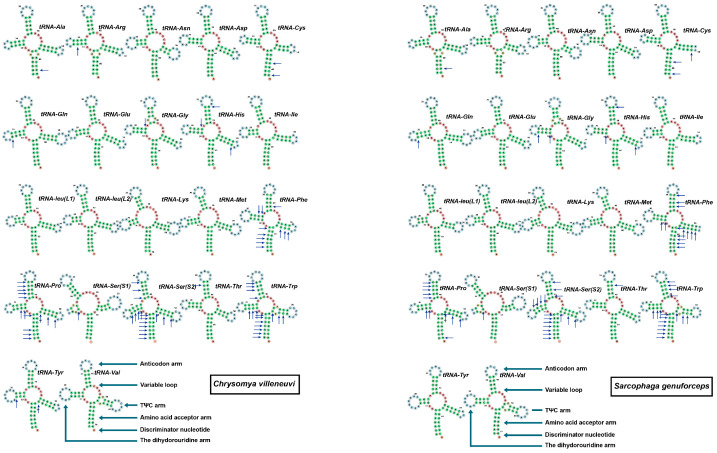
Predicted secondary cloverleaf structure for the tRNAs of *Chrysomya villeneuvi* and *Sarcophaga genuforcep.* The small blue arrow indicates the non-canonical base pair mismatches.

Visualization of the secondary structures of the 22 tRNAs (Figure 5) revealed that in both *C. villeneuvi* and *S. genuforceps*, *tRNA-Ser 1* lacks a dihydrouridine (DHU) arm and thus cannot form the typical cloverleaf secondary structure, instead forming a simple loop. The remaining 21 tRNAs adopt a complete cloverleaf structure. Besides typical A-U and G-C base pairing, both species show non-canonical base pair mismatches. In *C. villeneuvi*, the highest number of mismatches occur in *tRNA-Trp* (17), *tRNA-Ser2* (17), *tRNA-Pro* (14), and *tRNA-Phe* (11). Similarly, in *S. genuforceps*, mismatches are most frequent in *tRNA-Trp* (17), *tRNA-Ser2* (17), *tRNA-Phe* (14), and *tRNA-Pro* (12).

### 3.4. Phylogenetic Relationships

**Figure 6:**
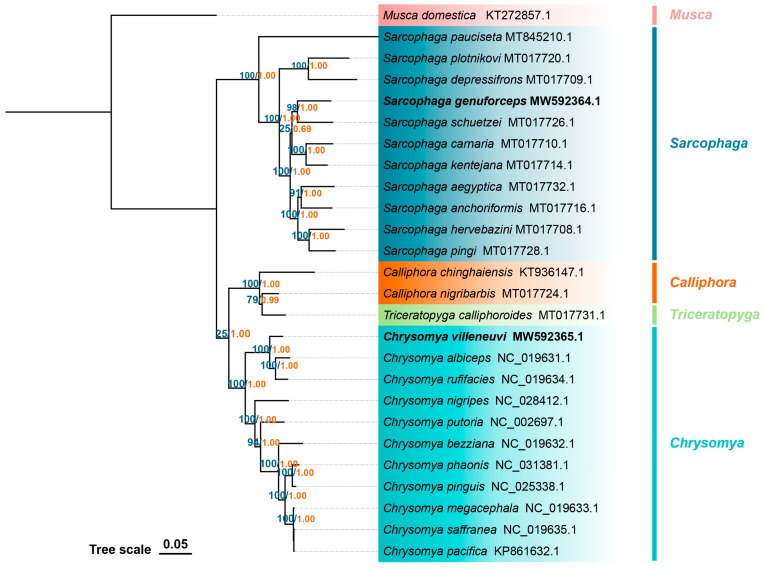
Maximum likelihood (ML) tree reconstructed based on the nucleotide sequences of 13 protein-coding genes (PCGs). The numbers around the nodes represent the bootstrap values from the ML analysis (BSs, left, in blue) and the posterior probabilities from the BI analysis (PPs, right, in orange).

The original tree files of ML and BI are displayed in the Supplementary Materials. As shown in Figure 6, the 11 species of *Chrysomya* and *Sarcophaga* each cluster distinctly into separate branches, with high node support value supporting the monophyly of both genera (*Chrysomya*: BS/BP = 100/1.00; *Sarcophaga*: BS/BP = 100/1.00). Within the *Chrysomya*, *C. villeneuvi* is more closely related to *Chrysomya rufifacies* and *Chrysomya albiceps*, with these three species forming a monophyletic group (BS/BP = 100/1.00) that is a sister to the remaining Calliphoridae species. In the *Sarcophaga*, *S. genuforceps* and *Sarcophaga schuetzei* are sister species (BS/BP = 98/1.00). However, the clade composed of *S. genuforceps* and *S. schuetzei* and the clade composed of *Sarcophaga carnaria* and *Sarcophaga kentejana* had lower node support values (BS/BP = 25/0.69).

**Figure 7:**
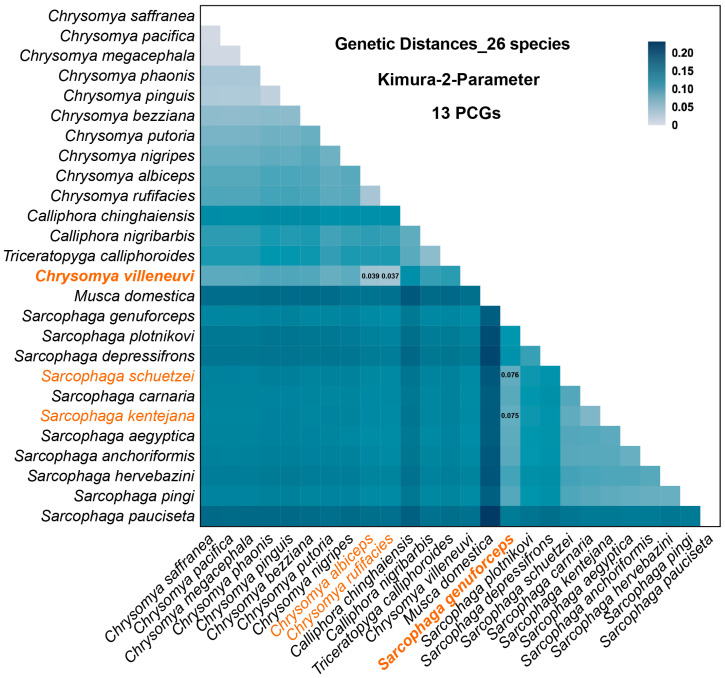
The genetic distance of the 26 species based on the 13 protein-coding genes (PCGs). The bold orange species represent those reported in this study, and the unbolded orange species denote closely related species.

The interspecific genetic distances calculated among 26 species are visualized in Figure 7. Consistent with the phylogenetic analysis, *C. villeneuvi* shows the closest genetic distances to *C. rufifacies* (0.037) and *C. albiceps* (0.039). Likewise, the closest genetic distances for *S. genuforceps* are to *S. kentejana* (0.075) and *S. schuetzei* (0.076), aligning with the relationships observed in the phylogenetic tree.

## 4. Discussion

The complete mitochondrial genome lengths of *C. villeneuvi* and *S. genuforceps* are 15,623 bp and 15,729 bp, respectively, falling within the typical mitochondrial genome size range of most insects (14–20 kb) [23,24,25]. As shown in Table 2, this variation in genome length is primarily attributed to differences in the length of the control region, consistent with patterns observed in other mitochondrial genomes [23,26]. Comparative analyses of mitochondrial genomes from the Sarcophaginae subfamily and the Calliphoridae family have demonstrated that their mitochondrial genomes are conserved in terms of gene content and arrangement, with a strong AT bias [23,27,28]. This study corroborates these findings, as the mitochondrial genomes of *C. villeneuvi* and *S. genuforceps* also exhibit high AT content, reflecting a pronounced AT bias. It has been widely recognized that most insect mitochondrial genomes share this characteristic, with adenine (A) content exceeding thymine (T), and guanine (G) content being lower than cytosine (C). This results in a positive AT-skew and a negative GC-skew, indicating strand asymmetry [27,29].

In the analysis of PCGs, despite *C. villeneuvi* and *S. genuforceps* belonging to different genera and families, their mitochondrial genomes display similarities to those of many metazoans [30,31], highlighting the conserved nature of insect mitochondrial genomes. *Chrysomya villeneuvi* and *S. genuforceps* utilize the stop codons TAG, TAA, and the incomplete stop codon T. These stop codons are commonly observed in insect mitochondrial genomes [32]. Ojala et al. [33] hypothesized that the incomplete stop codon T can be converted into a complete stop codon TAA through post-transcriptional polyadenylation.

The PCGs from the 11 Calliphoridae and 11 Sarcophagidae species analyzed in this study have undergone purifying selection throughout evolution, as indicated by Ka/Ks values of less than one for all PCGs. This suggests that these genes are highly conserved and may serve effectively as barcoding markers for species identification. In studies of sand flies, for example, both *cox1* and *cytb* genes have demonstrated utility in species identification, with *cox1* showing superior discriminatory power [2]. Additionally, *cox1* is widely regarded as the preferred DNA barcoding marker in forensic entomology and is extensively used for the molecular identification of various necrophagous insect species [34,35]. This is largely due to the gene’s slow conserved evolutionary rate in insects. In our study, *cox1* exhibited similarly low Ka/Ks values in both Calliphoridae and Sarcophagidae, approximately 0.02, underscoring its slow evolutionary rate and high conservation. These characteristics support its utility in distinguishing between Calliphoridae and Sarcophagidae.

In both *C. villeneuvi* and *S. genuforceps*, 21 of the 22 tRNAs formed typical cloverleaf secondary structures. The exception was tRNA-Ser (S1), which lacked the DHU arm and thus could not form a standard cloverleaf structure. The absence of the DHU arm in tRNA-Ser is highly prevalent in insect mitochondrial genomes and is considered an evolutionary adaptation to meet the compact structural and functional requirements of mitochondrial genomes. This phenomenon reflects the flexibility of mitochondrial genomes and highlights the unique structural and functional adaptations of insect mitochondrial tRNAs [13,29,36].

This study inferred the phylogenetic relationships of *C. villeneuvi* and *S. genuforceps* with other species based on phylogenetic analysis and genetic distances. It was found that *C. villeneuvi* is more closely related to *C. rufifacies* and *C. albiceps*. Similarly, B. Singh et al. [37] constructed a phylogeny of the *Chrysomya* genus using cytochrome oxidase subunit I and carbamoylphosphate synthetase DNA sequences (a total of 2386 bp). Their study revealed that *C. villeneuvi* is more closely related to *C. yayukae* (ML/BI = 100/1) and forms a sister clade with *C. rufifacies* and *C. albiceps* (ML/BI = 94/1). Morphology-based phylogenetics also indicates a high degree of morphological similarity among these three species. The larvae of *C. villeneuvi*, *C. rufifacies*, and *C. albiceps* are collectively known as “hairy maggots”, and distinguishing between the pupae of *C. villeneuvi* and *C. rufifacies* is particularly challenging [7,38,39].

For the subfamily Sarcophaginae, phylogenetic analyses of mitochondrial genomes have clarified the positions of *S. kentejana* and *S. schuetzei* within the subfamily, but no previous studies have addressed *S. genuforceps* [27,32]. This study provides the first insights into the evolutionary classification of *S. genuforceps* within the *Sarcophaga* genus, showing that it is closely related to *S. kentejana* and *S. schuetzei*. Information on *S. genuforceps* is extremely limited. Morphology-based phylogenetics places it in the subgenus *Bellieriomima*, while *S. kentejana* and *S. schuetzei* belong to the subgenera *Thyrsocnema* and *Kramerea*, respectively [9].

## 5. Conclusions

This study presents the first published mitochondrial genome data for *C. villeneuvi* and *S. genuforceps*, accompanied by a preliminary analysis. This study presents the first complete mitochondrial genome sequences of *C. villeneuvi* and *S. genuforceps*. These genomes exhibit characteristics consistent with other insect mitochondrial genomes, including conserved gene content and arrangement, high AT content, and strand asymmetry. The Ka/Ks values for all PCGs are less than one, indicating strong purifying selection, which underscores their conservation and utility as molecular markers for species identification. Phylogenetic analyses revealed that *C. villeneuvi* is closely related to *C. rufifacies* and *C. albiceps*, while *S. genuforceps* is more closely related to *S. kentejana* and *S. schuetze*. These findings provide valuable data and a solid foundation for further phylogenetic studies, species identification, and genetic analyses within the families Calliphoridae and Sarcophagidae.

## Figures and Tables

**Table 1:** Details of the species and mitogenomes used in this study.

Family	Genus	Species	Length (bp)	GenBank Accession Number
Calliphoridae	*Chrysomya*	*Chrysomya albiceps*	15,491	NC_019631.1
		*Chrysomya bezziana*	15,236	NC_019632.1
		*Chrysomya megacephala*	15,273	NC_019633.1
		*Chrysomya nigripes*	15,832	NC_028412.1
		*Chrysomya pacifica*	15,716	KP861632.1
		*Chrysomya phaonis*	15,831	NC_031381.1
		*Chrysomya pinguis*	15,838	NC_025338.1
		*Chrysomya putoria*	15,837	NC_002697.1
		*Chrysomya rufifacies*	15,412	NC_019634.1
		*Chrysomya saffranea*	15,839	NC_019635.1
		***Chrysomya villeneuvi***	**15,623**	**MW592365.1**
Sarcophagidae	*Sarcophaga*	*Sarcophaga carnaria*	14,952	MT017710.1
		*Sarcophaga kentejana*	14,943	MT017714.1
		*Sarcophaga schuetzei*	14,944	MT017726.1
		*Sarcophaga hervebazini*	14,939	MT017708.1
		*Sarcophaga pingi*	14,939	MT017728.1
		*Sarcophaga aegyptica*	14,943	MT017732.1
		*Sarcophaga anchoriformis*	14,943	MT017716.1
		*Sarcophaga depressifrons*	14,941	MT017709.1
		*Sarcophaga plotnikovi*	14,948	MT017720.1
		*Sarcophaga pauciseta*	16,035	MT845210.1
		***Sarcophaga genuforceps***	**15,729**	**MW592364.1**
Calliphoridae	*Calliphora*	*Calliphora nigribarbis*	16,241	MT017724.1
		*Calliphora chinghaiensis*	15,269	KT936147.1
	*Triceratopyga*	*Triceratopyga calliphoroides*	14,902	MT017731.1
Muscidae	*Musca*	*Musca domestica*	14,926	KT272857.1

**Table 2:** The composition and annotation of the mitochondrial genomes of *Chrysomya villeneuvi* and *Sarcophaga genuforceps*.

Gene	*Chrysomya villeneuvi*						*Sarcophaga genuforceps*		
	Strand (+/−: L/H)	Position	Size (bp)	Anticodon or Start/Stop Codons	Intergenic Nucleotides (bp)	Position	Size (bp)	Anticodon or Start/Stop Codons	Intergenic Nucleotides (bp)
*trnI*	+	1–66	66	GAT	−3	1–66	66	GAT	−3
*trnQ*	−	64–132	69	TTG	−1	64–132	69	TTG	−1
*trnM*	+	132–200	69	CAT	0	132–200	69	CAT	0
*nad2*	+	201–1217	1017	ATT/TAA	−3	201–1217	1017	ATT/TAA	−3
*trnW*	+	1215–1284	70	TCA	−9	1215–1284	70	TCA	−9
*trnC*	−	1276–1338	63	GCA	20	1276–1338	63	GCA	9
*trnY*	−	1359–1424	66	GTA	−2	1348–1413	66	GTA	−2
*cox1*	+	1423–2956	1534	TCG/T-	0	1412–2945	1534	TCG/T-	0
*trnL2*	+	2957–3022	66	TAA	5	2946–3011	66	TAA	6
*cox2*	+	3028–3715	688	ATG/T-	0	3018–3705	688	ATG/T-	0
*trnK*	+	3716–3786	71	CTT	−1	3706–3776	71	CTT	−1
*trnD*	+	3786–3852	67	GTC	0	3776–3842	67	GTC	0
*atp8*	+	3853–4017	165	ATT/TAA	−7	3843–4007	165	ATC/TAA	−7
*atp6*	+	4011–4688	678	ATG/TAA	−1	4001–4678	678	ATG/TAA	−1
*cox3*	+	4688–5476	789	ATG/TAA	6	4678–5466	789	ATG/TAA	6
*trnG*	+	5483–5547	65	TCC	0	5473–5537	65	TCC	0
*nad3*	+	5548–5901	354	ATT/TAA	2	5538–5891	354	ATT/TAA	1
*trnA*	+	5904–5968	65	TGC	−1	5893–5957	65	TGC	−1
*trnR*	+	5968–6031	64	TCG	0	5957–6020	64	TCG	0
*trnN*	+	6032–6097	66	GTT	0	6021–6086	66	GTT	0
*trnS1*	+	6098–6164	67	GCT	2	6087–6153	67	GCT	4
*trnE*	+	6167–6232	66	TTC	18	6158–6223	66	TTC	20
*trnF*	−	6251–6317	67	GAA	−1	6244–6310	67	GAA	−1
*nad5*	−	6317–8036	1720	ATT/T-	15	6310–8029	1720	ATT/T-	15
*trnH*	−	8052–8116	65	GTG	0	8045–8109	65	GTG	0
*nad4*	−	8117–9455	1339	ATG/T-	−7	8110–9448	1339	ATG/T-	−7
*nad4l*	−	9449–9745	297	ATG/TAA	8	9442–9738	297	ATG/TAA	2
*trnT*	+	9754–9818	65	TGT	−1	9741–9805	65	TGT	−1
*trnP*	−	9818–9885	68	TGG	1	9805–9872	68	TGG	1
*nad6*	+	9887–10,411	525	ATT/TAA	3	9874–10,398	525	ATT/TAA	−1
*cytb*	+	10,415–11,551	1137	ATG/TAA	0	10,398–11,534	1137	ATG/TAG	−1
*trnS2*	+	11,552–11,618	67	TGA	16	11,534–11,600	67	TGA	16
*nad1*	−	11,635–12,582	948	TTG/TAA	1	11,617–12,564	948	TTG/TAA	1
*trnL1*	−	12,584–12,648	65	TAG	3	12,566–12,630	65	TAG	0
*16S rRNA*	−	12,652–13,977	1326		0	12,631–13,956	1326		0
*trnV*	−	13,978–14,049	72	TAC	0	13,957–14,028	72	TAC	0
*12S rRNA*	−	14,050–14,835	786		0	14,029–14,814	786		0
Control region	+	14,836–15,623	788		0	14,815–15,729	915		0

**Table 3:** Nucleotide composition and skewness levels of the mitogenomes of *Chrysomya villeneuvi* and *Sarcophaga genuforceps*.

Species	Region	Size (bp)	Nucleotide Composition (%)						AT-Skew	GC-Skew
			A	T	G	C	A + T	G + C		
*Chrysomya villeneuvi*	PCGs	11,191	32.2	43.9	12.4	11.5	76.1	23.9	−0.15	0.04
	tRNAs	1469	37.8	38.5	13.3	10.5	76.3	23.8	−0.01	0.12
	rRNAs	2112	39.1	41.1	13.0	6.9	80.2	19.9	−0.02	0.31
	Control region	788	47.8	42.1	4.4	5.6	89.9	10.0	0.06	−0.12
	Whole mitogenome	15,623	39.7	37.7	9.3	13.3	77.4	22.6	0.03	−0.18
*Sarcophaga genuforceps*	PCGs	11,191	31.6	43.2	12.8	12.5	74.8	25.3	−0.16	0.01
	tRNAs	1469	37.9	38.1	13.5	10.5	76.0	24.0	0.00	0.13
	rRNAs	2112	39.0	41.3	12.8	6.9	80.3	19.7	−0.03	0.30
	Control region	915	47.7	40.7	5.0	6.7	88.4	11.7	0.08	−0.15
	Whole mitogenome	15,729	39.6	36.8	9.6	14.0	76.4	23.6	0.04	−0.19

## Data Availability

The annotated mitochondrial genome sequences were uploaded to NCBI and obtained GenBank accession numbers are MW592365.1 (*Chrysomya villeneuvi*) and MW592364.1 (*Sarcophaga genuforceps*).

## Supplementary Materials

The following supporting information can be downloaded at: https://www.mdpi.com/article/10.3390/insects16010026/s1. The supplementary materials include the original tree files from the ML and BI analyses.
