# Supplementary figures and images for: First Report and Phylogenetic Analysis of Mitochondrial Genomes of Chrysomya villeneuvi and Sarcophaga genuforceps
Source: Insects. 2024 Dec 29;16(1):26. doi: 10.3390/insects16010026 (PMC11766282; doi:10.3390/insects16010026)

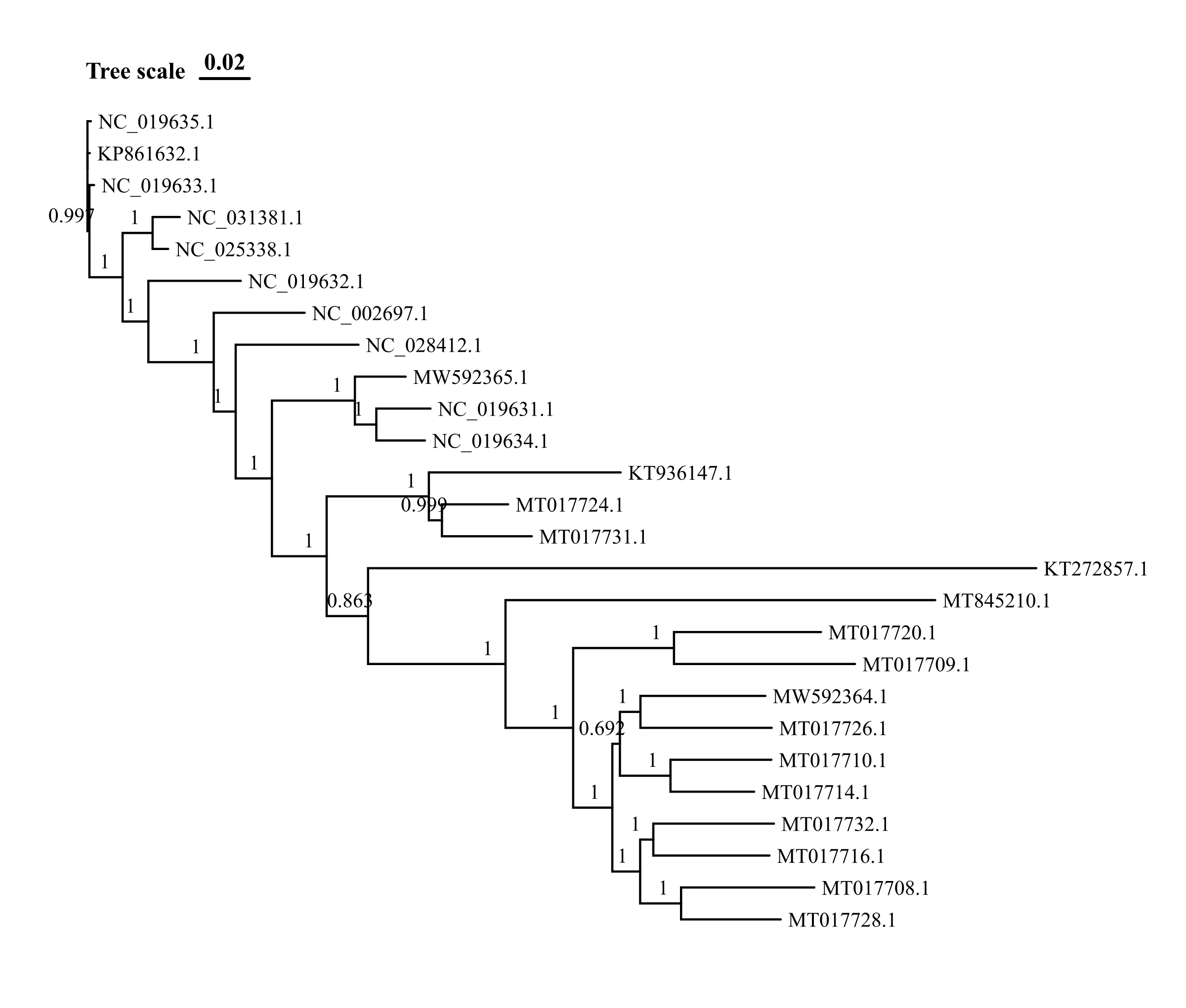

Supplement: Supplementary file 1 [file insects-16-00026-s001.zip › BI_original_no root.png]

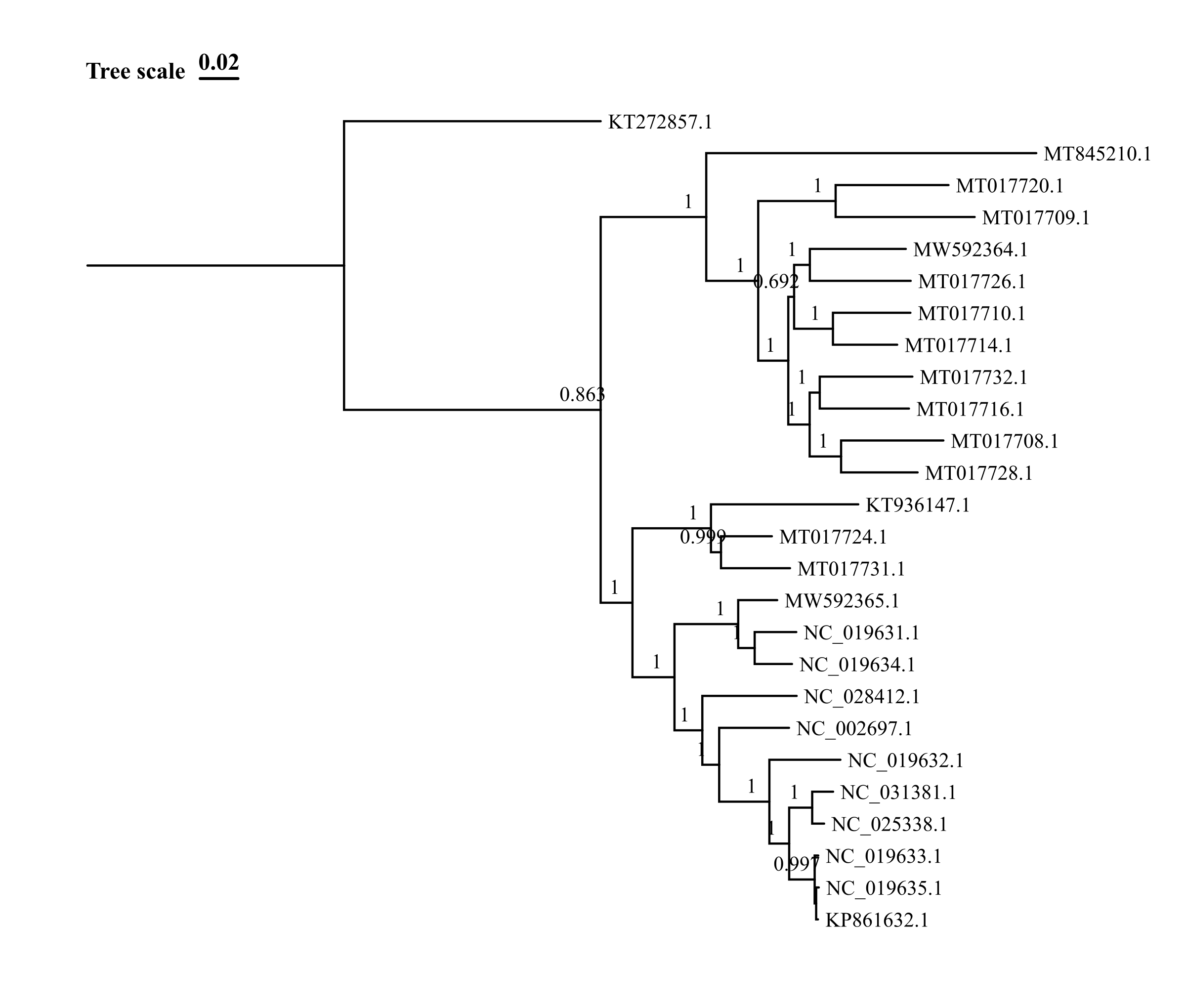

Supplement: Supplementary file 1 [file insects-16-00026-s001.zip › BI_original_re toot.png]

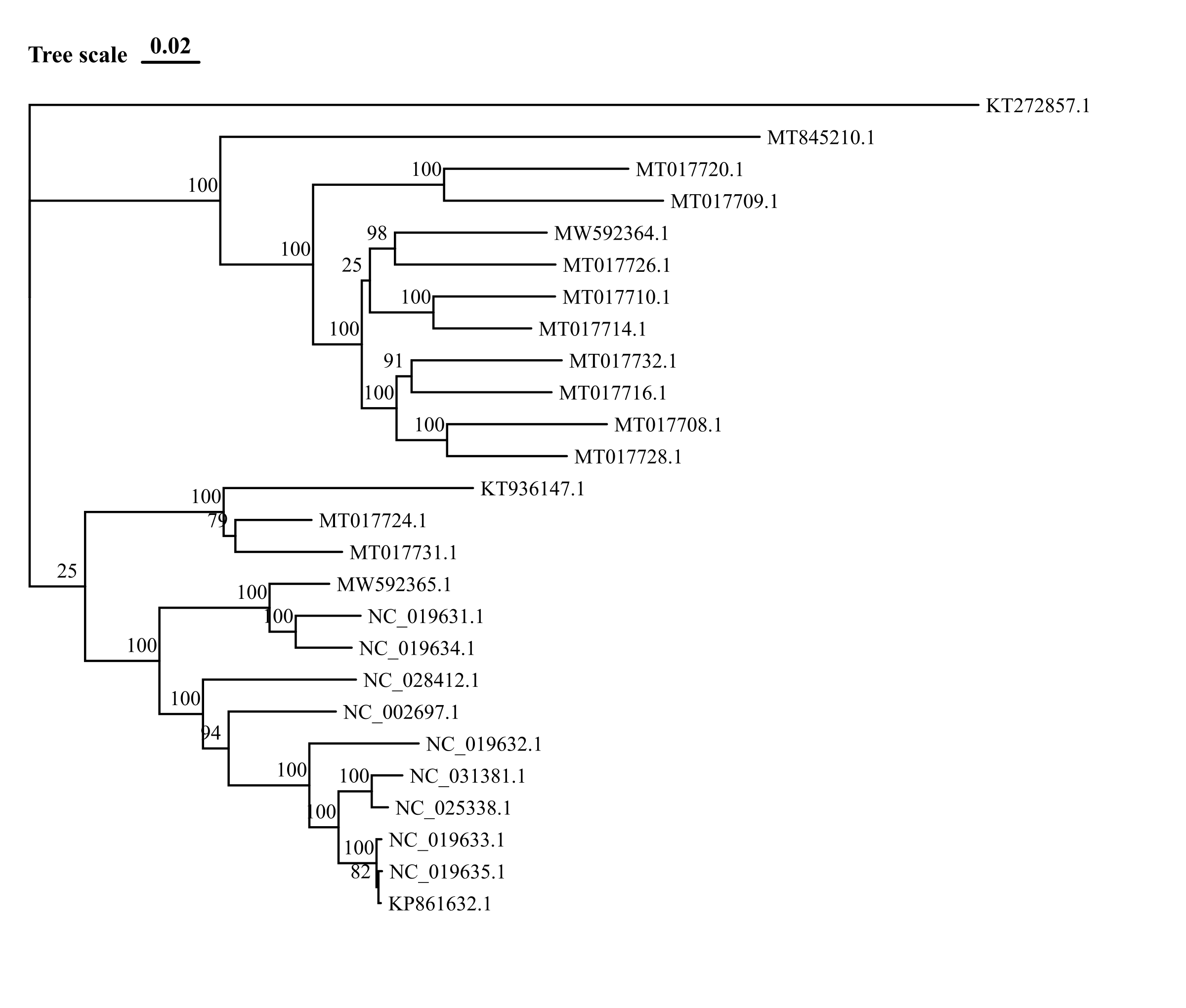

Supplement: Supplementary file 1 [file insects-16-00026-s001.zip › ML_original_no root.png]

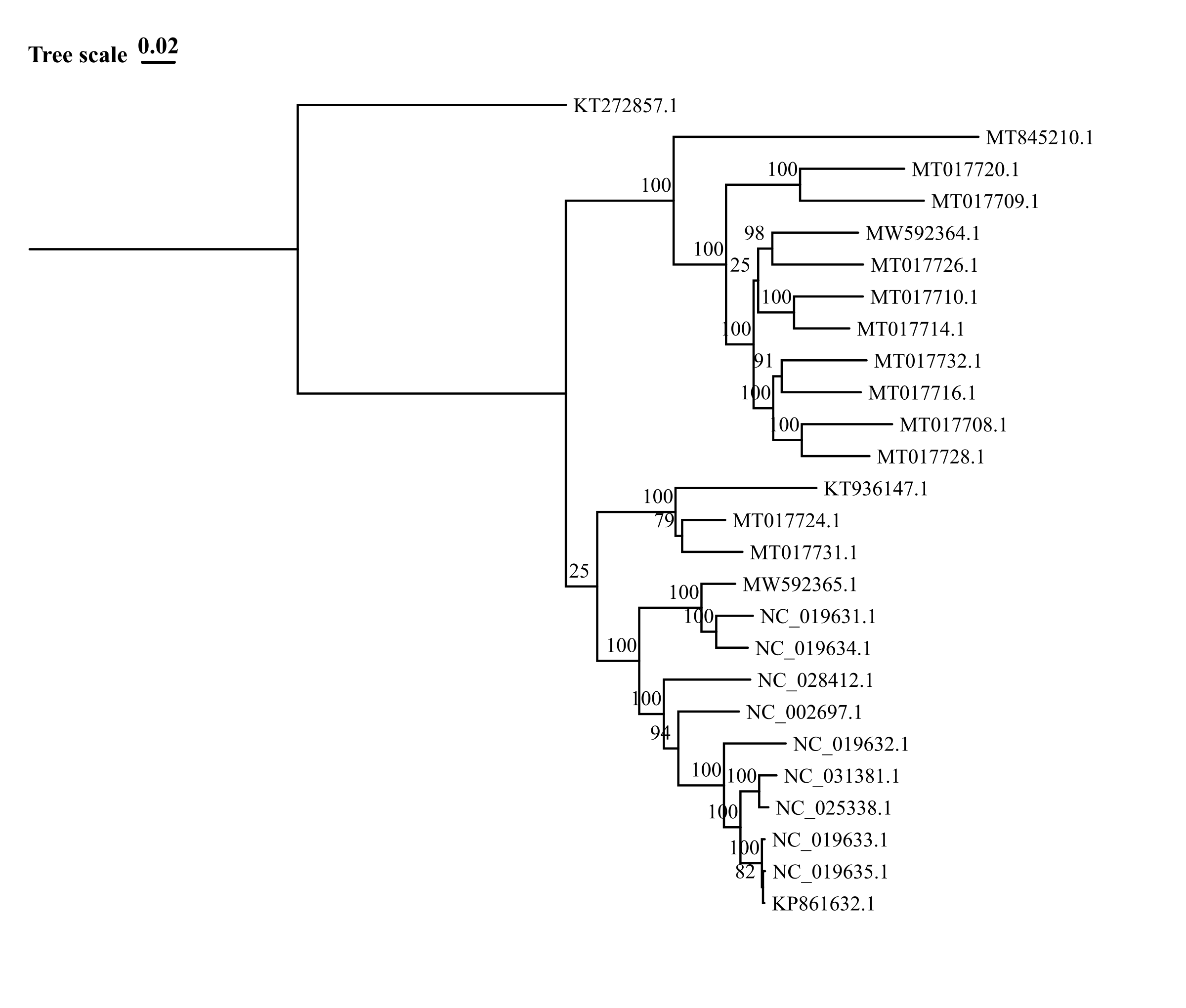

Supplement: Supplementary file 1 [file insects-16-00026-s001.zip › ML_original_reroot.png]
